# Salivary testing of COVID-19: evaluation of serological testing following positive salivary results

**DOI:** 10.1186/s12879-021-06108-5

**Published:** 2021-05-04

**Authors:** Lisa Caulley, Julie Shaw, Martin Corsten, Nadia Hua, Jonathan B. Angel, Guillaume Poliquin, Jonathan Whelan, Kym Antonation, Stephanie Johnson-Obaseki

**Affiliations:** 1 Department of Otolaryngology – Head and Neck Surgery, University of Ottawa, 501 Smyth Rd, Ottawa, K1H8L1 Canada; 2 Department of Pathology and Laboratory Medicine, University of Ottawa, 451 Smyth Road, Ottawa, K1H8L1 Canada; 3 Division of Otolaryngology – Head and Neck Surgery, Dalhousie University, 6299 South St, Halifax, B3F4R2 Canada; 4 Division of Infectious Diseases, University of Ottawa, 451 Smyth Road, Ottawa, K1H8L1 Canada; 5 Chronic Disease Program, Ottawa Hospital Research Institute, 1053 Carling Ave, Ottawa, K1H8L1 Canada; 6 National Microbiology Laboratory, Public Health Agency of Canada, 1015 Arlington St, Winnipeg, R3E3M4 Canada; 7 Department of Pediatrics & Child Health, University of Manitoba, 66 Chancellors Cir, Winnipeg, R3T2N2 Canada; 8 Department of Undergraduate Medical Education, Faculty of Medicine, University of Ottawa, 451 Smyth Road, Ottawa, K1H8L1 Canada

**Keywords:** Coronavirus, COVID-19, SARS-CoV-2, Antibodies, Saliva

## Abstract

**Background:**

Salivary detection of severe acute respiratory syndrome coronavirus 2 (SARS-CoV-2) has been proposed as an alternative to nasopharyngeal or oropharyngeal swab testing. Our group previously published a study demonstrating that both testing methods identified SARS-CoV-2 using polymerase chain reaction (PCR)-based detection methodology. We therefore conducted a follow-up study using antibody testing to evaluate the accuracy of saliva versus swabs for COVID-19 detection and the durability of antibody response.

**Methods:**

Venous blood samples were collected from consenting participants and the presence of serum antibodies for SARS-CoV-2 was evaluated on a large, automated immunoassay platform by the Roche anti-SARS-CoV-2 qualitative assay (Roche Diagnostics, Laval Quebec). Individuals with a serum antibody cut-off index (COI) ≥ 1.0 were considered positive.

**Results:**

In asymptomatic and mildly symptomatic patients with a previously positive standard swab and/or saliva SARS-CoV-2 PCR-test, 42 demonstrated antibodies with 13 patients positive by swab alone, and 8 patients positive by saliva alone.

**Conclusions:**

Despite their status as ‘current standard’ for COVID-19 testing, these findings highlight limitations of PCR-based tests.

## Background

Salivary detection of severe acute respiratory syndrome coronavirus 2 (SARS-CoV-2) has been proposed as an alternative to nasopharyngeal or oropharyngeal swab testing. Our group recently published a study of 1939 paired swab and saliva samples in an ambulatory testing center, which demonstrated that both testing methods identified SARS-CoV-2 using polymerase chain reaction (PCR)-based detection methodology [1]. However, there was discordance between saliva and swab testing in approximately 30% of samples. Our findings aligned with recent studies that have likewise supported the utility of saliva samples for detection of COVID-19, with the same caveats regarding discordance [2–8]. We conducted a follow-up study using antibody testing to evaluate the accuracy of saliva versus swabs for COVID-19 detection and the durability of antibody response.

## Objective

To evaluate humoral response in participants that tested positive for SARS-CoV-2 on saliva and/or swab testing.

## Methods

Following research ethics board approval, asymptomatic and mildly symptomatic individuals who tested positive for SARS-CoV-2 on saliva, and/or swab testing at a COVID-19 testing center in Ottawa, Canada were invited to participate in this follow-up study. Swab and/or saliva test PCR analysis needed to be positive for the SARS-CoV-2 envelope (E) gene, at minimum, for participants to be included in this follow-up study. Saliva samples with an E assay cycle threshold (CT) value greater than 37, were repeated and followed by confirmation with an assay to detect RNA-dependent RNA polymerase (RdRp) gene. Methodological details of the initial testing are published elsewhere [1]. Participants were contacted by telephone to provide verbal consent for study participation. Venous blood samples were collected at The Ottawa Hospital and analyzed at the Eastern Ontario Regional Laboratory Association. Presence of antibodies for SARS-CoV-2 was evaluated on a large automated immunoassay platform by the Roche anti-SARS-CoV-2 qualitative assay (Roche Diagnostics, Laval Quebec). Individuals with a serum antibody cut-off index (COI) ≥ 1.0 were considered positive [9]. It should be noted that given the qualitative nature of the assay, the magnitude of the COI value above the cut-off does not reflect the amount of antibody present in the sample. The target protein used in the assay was the nucleocapsid of the SARS-COV-2 virus, which by detecting antibodies against the nucleocapsid is not sero-type specific. The assay used 12 uL of serum for analysis and did not require protein extraction. Positive and negative controls were analyzed with each batch of specimens. The control samples were sourced from known COVID positive patients (positive control) and negative (negative control, i.e., COVID-naïve patients, collected prior to December 2019).

## Results

**Fig. 1:**
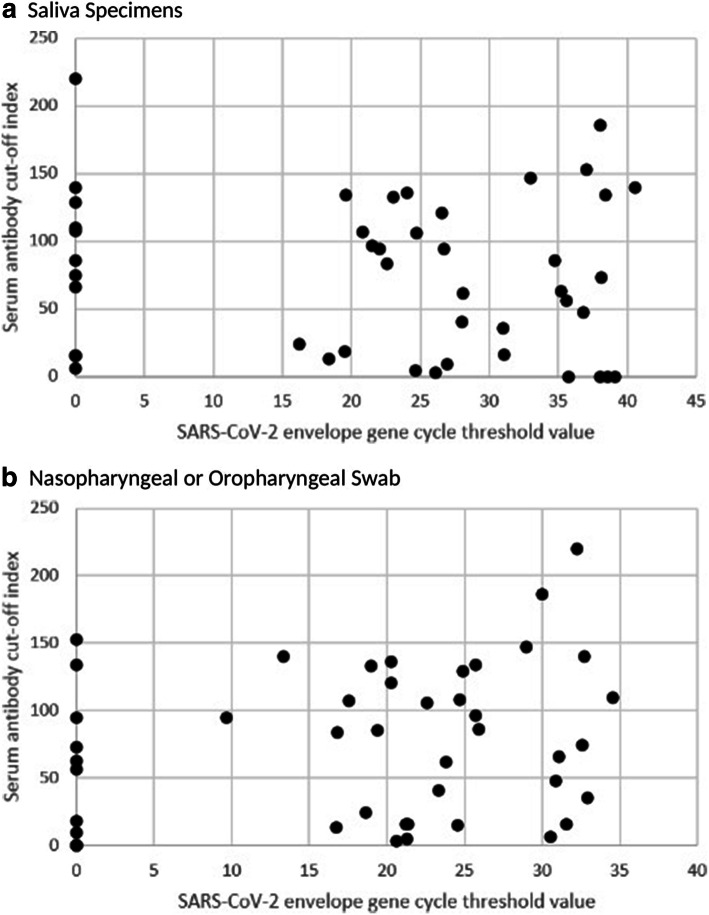
SARS-CoV-2 Antibody Titers in Saliva Specimens and Nasopharyngeal or Oropharyngeal Swab Specimens. SARS-CoV-2 - severe acute respiratory syndrome coronavirus 2

Of the 70 patients that initially tested positive, 46 consented to participate. 74% of participants were female, and median age was 42.5 (Range 20–72 years old). The median time from positive PCR to antibody analysis was 144.5 days (IQR: 131.5–153.5 days). Of these 46 patients consenting to the study and thereby having a previously positive swab, saliva or both SARS-CoV-2 test, 42 (91.3%) demonstrated SARS-CoV-2 antibodies (Table 1, Fig. 1). Of these 42 participants with positive serum antibodies, 21 had been positive by both swab and saliva (Median Age 43 (20–67), 71% female), 13 previously tested positive by swab alone (Median Age 44 (33–72), 85% female), and 8 were participants that previously tested positive by saliva alone (Median Age 43 (29–61), 50% female).

**Table 1:** SARS-CoV-2 test results of study participants

	SARS-CoV-2 Antibodies Detected	SARS-CoV-2 Antibodies Not Detected
Swab Positive	34	0
Swab Negative	8	4
Saliva Positive	30	4
Saliva Negative	12	0

Swab: Nasopharyngeal or oropharyngeal swab

*SARS-CoV-2* severe acute respiratory syndrome coronavirus 2

## Discussion

We compared the presence of serum SARS-CoV-2-specific antibodies in patients that had previously tested positive on saliva and/or swab RT-PCR analysis. We observed an antibody detection rate of 91% in this population of high-risk asymptomatic or mildly symptomatic patients.

Despite their status as the ‘current standard’ for COVID-19 testing, PCR-based tests have limitations. In our study, we identified antibodies in patients who tested negative by swab or saliva PCR. These findings may highlight false negative PCR-tests, subsequent infection with SARS-CoV-2 or false positive antibody tests. Furthermore, we observed patients with no detectable antibodies despite a positive initial PCR-based test. This only occurred in four patients positive by saliva alone and negative on swab testing; potentially indicating false positive saliva tests, or chance occurrence.

Of the four individuals that were antibody negative, three were positive by E gene with a CT value above 35, and a negative RdRp gene. This may support the use of a second gene target to be certain that a high CT value of one gene accurately represents a positive result. Antibodies were not detected in one participant despite two positive gene targets, which may represent the absence of antibody response with mild COVID-19 disease severity, the loss of antibody response over time or laboratory error. However, given the small sample size, these results must be interpreted with caution. Furthermore, it should be noted that a direct association between PCR positivity and seroconversion should be interpreted cautiously, as PCR positivity may also be due to shedding of non-viable virus or non-infectious genome fragments in asymptomatic people [10].

Our findings are relevant given the ongoing uncertainty surrounding the durability of SARS-CoV-2 antibodies [11–13]. We observed sustained seropositivity in over 90% of mildly symptomatic or asymptomatic individuals with median follow-up 5 months after illness presentation. This contrasts a recent report that suggests a rapid decline in antibodies over time, particularly in asymptomatic individuals [13]. Further research is therefore required to continue exploring the magnitude and duration of IgG responses in patients recovered from SARS-CoV-2 infection. Of note, the SARS-CoV-2 antibodies detected in these participants were directed against the nucleocapsid of the virus. These are not neutralizing antibodies and therefore do not imply immune protection.

Importantly, our study had limitations. First, over 30% of eligible patients declined participation. Second, there remains no true “gold standard” for SARS-CoV-2 detection in evaluation of salivary testing. Third, the Roche serology assay has the potential to produce both false positives and false negatives in detecting previous COVID-19 infection [9].

In this study, humoral immune response to SARS-CoV-2 was observed in over 90% of ambulatory participants that demonstrated positivity on swab, saliva, or both specimens. Participants remained seropositive 5 months post-infection. These findings also highlight the interpretative limitations with salivary results in the setting of single-targeted gene PCR, high CT values, and mild disease severity.

## Data Availability

The datasets used and/or analysed during the current study are available from the corresponding author on reasonable request.
